# Intelligence

**Published:** 1807-08

**Authors:** 


### INTELLIGENCE.

The *Senatus Academicus* of the University of *Edinburgh*, has conferred the degree of Doctor in Medicine on the following Gentlemen, after having gone through the appointed examinations, and publicly defended their inaugural dissertations.

OF SCOTLAND.

| | |
|---|---|
| Joshua Henry Davison, | De Cynanche Tracheali. |
| Robert Tytler, | De Oxygenio. |

FROM ENGLAND.

| | |
|---|---|
| Henry Fearon, | De Dysenteria. |
| Joseph Ayre, | De Temperaturæ Effectibus. |
| Albert Perry Moss, | De Apoplexia. |
| Thomas Bent, | De Hepatitide Chronica. |
| David Plenderleath, | De Pneumonia. |
| George Green Spilsbury, | De Phthisi Pulmonali. |
| John Thomson, | De Chorea. |
| John Armstrong, | De Causis Morborum Hydropicorum. |
| John M. B. Pigot, | De Morbis Ebriosorum. |
| Richard Maddock Hawley, | De Fonte Caloris. |
| Edward Parker Charlesworth, | De Scarlatina. |
| Robert Gooch, | De Rachitide. |
| William Shearman, | De Pneumonia. |
| W. Brackenbury Bousfield, | De Hydrocephalo Acuto. |
| Henry Snow, | De Rheumatismo Acuto. |
| Baldwin Wake, | De Typhi Remediis. |
| Samuel Liston, | De Calculo. |
| Joshua Ireland, | De Mania. |

FROM IRELAND.

| | |
|---|---|
| William Hudson, | De Rheumatismo Acuto. |
| William Stott, | De Hepatitide Acuto. |
| Robert Nixon, | De Voce Humana. |
| Peter Gilroy, | De Ictero. |

David

| | |
|---|---|
| David Barry, | De Phlegmasia Dolente Puerperarum. |
| Joseph Brownlow, | De Sanguinis Circulatione. |
| John Pigot, | De Principio Vitali. |
| Henry Mc Creery, | De Pneumonia. |
| Albert H. Callanan, | De Ascite. |
| Stephen Laur. O'Brien, | De Asthmate Spasmodico. |
| Eugene O'Neill. | De Hernia Inguinali. |
| Daniel Linchan, | De Respiratione. |
| Samuel Robinson, | De Pneumonia. |

FROM PORTUGAL.

| | |
|---|---|
| Francis de Souza, | De Cataracta. |

FROM AMERICA.

| | |
|---|---|
| James Low, | De Tetano. |

FROM THE WEST INDIES.

| | |
|---|---|
| Thomas Kiniaid Wint, | De Amenorrhœa. |
| Benjamin Hardtman, | De Aure Humana. |
| Charles Farquharson, | De Phthisi Pulmonali. |

---

Dr. FRYDENBERG, of Iceland, has sent to the Society of Rural Economy of Copenhagen, a Memoir on the possibility of converting the *scetang* (a marine plant which abounds in the sea that washes the shore of that island, and other parts of the Danish dominions) into food fit for man. If the means he proposed for this purpose should prove successful, he will have the satisfaction of conferring an inestimable benefit on Iceland; a country so ill provided with alimentary productions, that all the precautions of Government are sometimes found ineffectual to preserve the inhabitants from the horrors of famine. Hitherto the *scetang* has been used in Denmark only for fuel, and in Norway for making kelp.

M. REDOWSKI, who had been nominated Botanist to the projected embassy from the court of Russia to China, is engaged in a very extensive botanical tour, at the Emperor's expence, thro' the most remote north-eastern districts of Asia, including the islands between that continent and Japan to the southward, and the coast of North America to the eastward. He will be accompanied by a Mathematician, who is to make astronomical observations.

We have great satisfaction in announcing the humane, liberal, and honourable conduct of the Medical Practitioners of the City, and County of Worcester, respecting Vaccination. We trust that the example will be followed by every County, City, and Town in the United Kingdom:—not excepting LONDON.

Extract from the Worcester Journal, March 26, 1807.

We the undersigned, convinced, by the experience of many years, of the great and numerous advantages resulting from the *Inoculation of the Cow-pox*, both as it regards the preservation and happiness of individuals, and the interests of society, and which becomes become more striking, when they are contrasted with the evils that unavoidably attend the inoculation of so contagious and dangerous a disease as the SMALL-POX; feel ourselves called upon to enter into the following RESOLUTION:

"We will not, under any circumstances or application whatever, inoculate, or sanction the inoculation for the SMALL-POX."

This resolution is undersigned by Cameron, Wilson, Barnet, Deane, M. D. D. —Cole, Sandford, Carden, Lambe, Rayment, Wilmore, Nash, Hill, Hebb, Romney, Wheeler, Blower, Ricketts, Jukes, Doughty, Horniblow, Smith, Wainwright, Shaw, Geust, T. Wainwright, Moss, Radley, Short, Cartwright, and F. Smith. It is also announced in the same paragraph, that "the surgeons of the WORCESTER INFIRMARY, continue to *vaccinate the poor gratis*, every Monday and Thursday morning, at the Infirmary.

The Small-pox has lately occasioned dreadful mortality in those parts of Arabia, bordering on the Persian Gulph. The son of SHEIK RAMAH was among the victims. The British Resident has profited by the alarm resulting from this fatal visitation, to introduce Vaccination. All the principal natives submitted to the process.

The same disease also raged last year throughout Malacca, with a virulence before unknown; not a single instance occurred of a recovery, when the infection had been caught in the natural mode. Dr. Loftie, the Garrison Surgeon, saved many lives, and has since very generally introduced the vaccine process, the natives flocking to him from all parts to be inoculated.

Among the numerous Societies and Institutions established in the metropolis, for the alleviation of diseases and inabilities among the poor, the *new societies* for relieving the diseases of the eye and ear, and the treatment of ruptures, deservedly excite very general attention, on account of the great and numerous benefits they confer on this class of invalids. A false modesty too often prolongs the sufferings, and not unfrequently proves the destruction of the ruptured of all ranks. The establishment of popular societies for the relief of these complaints, will have a powerful effect in banishing this false and dangerous delicacy, and thereby of saving many valuable lives that would otherwise be lost, or continue useless to society.

A Correspondent hints that we are often baffled in attempting to produce mercurial action in the system by the internal exhibition of mercury, on account of its proneness to disorder the bowels. He suggests that a free use of opium from the first will never fail to obviate this inconvenience; and as we are *compelled* on many occasions to resort to this internal mode of employing this specific, he deems it of considerable importance to be able to guard against disappointments.

Mr. PARKES has for some time been engaged in preparing a new edition of the Chemical Catechism. It will contain an account of all the new discoveries, with other very considerable additions and improvements.

At

Holland, a country where Medicine has been taught with such success, does not possess any work, the object of which is to apply physical science to the interpretation of the laws. For the purpose of supplying this desideratum, Dr. KEGLELOOT, a celebrated physician, has been engaged during the last four years in collecting materials from all the medical writers who had peculiarly devoted their talents to this branch of study, such as Frank, Arneman, Plenck, in Germany; Foderé and Mahon, in France; Vesace and Cardile, in Italy, &c. As the work is chiefly intended for the learned, the Doctor has written it in the Latin language. From the plan and known abilities of the Author, and the luminous developement of all the parts of his subject, it is confidently expected that it cannot fail meeting with the approbation of the Dutch government, as the most public and complete Treatise that has yet appeared on Medical Jurisprudence.

---

M. VAUQUELIN, Professor of Chemistry in the Museum of Natural History at Paris, has analyzed various Specimens of Ore from the famous Silver Mine of Guadal Canal, in Estremadura, and discovered in them platina, united with silver, copper, iron, antimony, arsenic, lead and sulphur, sometimes amounting to one tenth of the mass.

---

In the course of 1806, among the deaths in the extensive empire of Russia, there were

| | | | | | | |
|---|---|---|---|---|---|---|
| 1 | between | 145 | and | 150 | years of age. |
| 1 | —— | 130 | and | 135 | —— |
| 4 | —— | 125 | and | 130 | —— |
| 6 | —— | 120 | and | 125 | —— |
| 32 | —— | 115 | and | 120 | —— |
| 26 | —— | 110 | and | 115 | —— |
| 86 | —— | 105 | and | 110 | —— |
| 137 | —— | 100 | and | 105 | —— |
| 1134 | —— | 95 | and | 100 | —— |

---

Dr. YOUNG, who has just published a Course of Lectures on Natural Philosophy, delivered at the Royal Institution, has begun to collect Materials for a Work, in a form nearly similar, relating to every Department of Medical Knowledge; it will be comparatively more concise than the above Lectures, in proportion to what has been written respecting Physic; but much more complete with regard to all that is known with certainty, and can be applied with utility.

---

Dr. CHEYNE is about to publish a Dissertation on *Hydrocephalus acutus*, or Dropsy in the Brain, illustrated by a Series of Clinical Cases and Dissections.

Dr. B. S. Barton, Professor of the Materia Medica in the University of Pennsylvania, is about to publish Elements of Zoology; or, Outlines of the Natural History of Animals.

---

An Experimental Inquiry into the Chemical and Medical Properties of the *Statice Lemonium* of Linnæus, has lately been published at New York, by Valentine Mott, President of the American Orsculapian Society.

---

Dr. Hosack, Professor of Botany and the Materia Medica in Columbia College, and F. R. S. of London, has recently printed a Catalogue of Plants contained in the Botanic Garden at Elgin, near New York.

---

At Edinburgh, in the beginning of November, Mr. Walker will commence a Course of Lectures on Physiology, or the Laws of Organic Existence.

---

The following useful Plan for VILLAGE or PARISH LIBRARIES has been circulated by some public-spirit ed Individuals, and as many of our Country Readers may have Opportunities to promote such Establishments, we do not consider it wholly foreign to the Business of our Journal to give it a Place.

"It is proposed to establish in every village or parish in the kingdom a small Library, consisting chiefly of Books of Agriculture, History, Modern Voyages and Travels, and other subjects of rational instruction and general utility.

"The Funds for commencing and maintaining such a Library, to be raised by a subscription of five shillings per quarter for three years, and of a half crown per quarter afterwards.

"The resident Clergyman, for the time being, to be President of the Society, and a Treasurer to be appointed annually from among the subscribers.

"The subscriptions to be received, the accounts to be kept, and the books to be circulated and registered by the Parish Clerk, or by the Parish Schoolmaster, who, besides having the use of the books for his own reading, is to be entitled to the fines.

"The books to be kept in the Vestry Room, at the house of the officiating clergyman, or at any other convenient place, in a room which shall be accessible to the subscribers.

"Quarterly meetings to be held of the subscribers at the place where the books are kept, when new books are to be ordered, accounts stated, and regulations formed.

"No book to be kept for reading more than a month, under the forfeiture of one penny per day afterwards; and no Magazine, Review, or pamphlet, to be kept more than five days under a similar penalty.

"The

"The first object of such a Society, should be to possess itself of the County Reports, and other Books published by the Board of Agriculture, of Gregory's Cyclopedia, some of Arrowsmith's Maps, Dickson's Agriculture, a System of Geography, Mavor's Universal History, Johnson's Dictionary, and Hume and Belsham's (the last revised edition) History of England. It should also begin to take in for periodical circulation, the Monthly Magazine, the Annals of Agriculture, the Oxford Review, and the Journal of Modern Voyages and Travels.

"The Library to be considered as the property of the subscribers, and of their resident heirs or successors, as long as they shall contiuue to pay their quarterly contributions within twelve months after they fall due; but any parishioner, may, at any time, be at liberty to become a reader of the library on paying three shillings for a single quarter.

"N. B. To establish such a Library, it seems only to be requisite that a fair copy of this plan should be affixed to the church door, that the Clergyman, or Parish-clerk should solicit the names of the chief parishioners; and as soon as a dozen have paid their first subscription, the Society must be considered as formed.—Should any Nobleman or Gentleman lend his countenance to the plan, and contribute a donation of ten or twenty pounds, its establishment could scarcely fail to be permanent."

